# Perceived stigma by children on antiretroviral treatment in Cambodia

**DOI:** 10.1186/s12887-014-0300-9

**Published:** 2014-12-10

**Authors:** Hubert Barennes, Sovann Tat, Daniel Reinharz, Ung Vibol

**Affiliations:** Agence Nationale de Recherche sur le VIH et les Hepatites, Preah Monyvong 5 Blvd, Phnom Penh, Cambodia; Institut Francophone pour la Médecine Tropicale, Vientiane, Vientiane, Lao PDR; ISPED, Centre INSERM U897-Epidemiologie-Biostatistique, Univ. Bordeaux, F-33000 Bordeaux, France; Epidemiology Unit, Pasteur Institute, Phnom Penh, Cambodia; Stung Treng Regional Training Center for Nursing and Midwifery Cambodia, Stung Treng, Cambodia; Département de médecine sociale et préventive, Université Laval, Québec, Canada; University of Health Science, Phnom Penh, Cambodia

**Keywords:** AIDS, Cambodia, Children, Discrimination, Exclusion, HIV, Perceived stigma, School, Stigma, Stigmatization

## Abstract

**Background:**

HIV-related stigma diminishes the quality of life of affected patients. Little is known about perceived and enacted stigma of HIV-infected children in resources-limited settings. We documented the prevalence of perceived stigma and associated factors associated among children on antiretroviral therapy (ART) at a referral hospital in Cambodia.

**Methods:**

After informed consent, a standardized pre-tested 47-item questionnaire was confidentially administered to consecutive children (7 to 15 years) or their guardians if the child was 18 months to 6 years, during their routine ART visits. The questionnaire explored the sociodemographics of the child and the parents, HIV history, adherence to ART, tolerance of ART and perceived stigma. Associations between perceived stigma and the children’s characteristics were measured by bivariate and multivariate analyses.

**Results:**

Of 183 children, 101 (55.2%) had lost at least one and 45 (24.6%) both parents; 166 (90.7%) went to school. Of 183 children (female: 84, 45.9%, median age 7.0 years, interquartile range: 2.0-9.6), 79 (43.2%) experienced perceived stigma, including rejection by others (26.8%), no invitations to social activities (18.6%) and exclusion from games (14.2%). A total of 43 (23.5%) children were fearful of their disease and 61 (53.9%) of 113 older than 6 years reported knowledge of their HIV status. Of 136 children over five years and eligible for education, 7 (3.8%) could not go to school due to perceived stigma. Incomplete adherence to ART was reported for 17 (9.2%) children. In multivariate analysis, school attendance (odds ratio [OR]: 3.9; 95% confidence interval [CI]: 2.0-7.9) and income of less than one dollar per person per day (OR: 2.2, 95% CI: 1.1-4.5) were associated with perceived stigma. Conversely, receipt of social support (OR: 0.4, 95% CI 0.2-0.9) was associated with lower risk of perceived stigma.

**Conclusion:**

Perceived stigma in pediatric ART patients remains a significant issue in Cambodia. Psychological support and interventions should be developed in hospitals, schools, and underprivileged communities to prevent HIV-related stigma for affected children.

## Background

Stigma and discrimination associated with HIV and AIDS are complex concepts that result in a “process of devaluation” or “loss of status and discrimination” for people either living with or associated with HIV and AIDS [1]. Stigma contributes to the “hidden burden of disease” [2]. Discrimination refers to the unfair and unjust treatment of an individual based on his/her real or perceived HIV status [1]. Different types of HIV/AIDS stigma have been described: perceived stigma, enacted stigma, internalized stigma, and associative stigma [1,3]. Perceived stigma corresponds to “felt” stigma and refers to all types of stigmatizing attitudes or behavior towards People Living with HIV/AIDS (PLWHA), as experienced or perceived by themselves or others. Enacted stigma refers to the real experience of discrimination experienced by the target of stigma. Internalized stigma involves thoughts and behavior stemming from a person’s own negative perceptions about himself/herself because of their HIV status [1,3]. Associative stigma (also called secondary stigma) refers to stigma that results from a person’s association with PLWHA [3]. Stigma may be perceived and enacted at various levels; individual, family, institutions and society.

Stigma has important implications related to misconceptions of the disease. This may lead to various forms of serious discrimination and exclusion for children, including lack of access to, or expulsion from school, involuntary separation from parents, being denied housing, and for adults having to pay extra rent, unemployment, isolation and even punishment [1,4-6]. Internalised stigma felt by people living with HIV can, when combined with feelings of isolation, lead to depression, self-imposed withdrawal and even suicide.

Stigma undermines the success of prevention programs and can negatively influence access to antiretroviral therapy (ART), diminishing its effectiveness [7] or may affect the quality of life for people undergoing ART [8]. HIV-related stigma discourages individuals who are aware of their HIV-positive status from sharing information about their disease with sexual partners, making it difficult to prevent the spread of the infection or to plan for the future of their surviving children and family members [9]. Few parents are ready to disclose their HIV status or to test their children for fear of stigma [10]. Stigma has been evoked by mothers as a major reason for discontinuing ART after weaning their child [11]. Moreover, in high HIV prevalence and HIV cluster areas secondary stigma may extend to non-infected members of the community [12].

Children living with HIV or children living with PLWHA are particularly prone to being stigmatized by the community and having psychological problems [13,14]. However, the extent of HIV-related stigma, and its implication on children’s health and psychological outcomes have rarely been documented in developing countries and stigma measurement scales differ according to studies [3,4,14-16].

In Cambodia, since the detection of the first HIV case in 1991, the prevalence has diminished from 1.3% in 2003 to 0.8% in 2011[17,18]. In 2010, there were 8512 children living with HIV in the country [18].

The *People Living with HIV Stigma Index Cambodia* study was conducted in 2010 among 397 adults (71.3% women) [19]. The survey revealed various levels of stigma manifestations and discrimination among adult PLWHA. The main manifestations included gossip (37.6%), manipulation and psychological pressure (33.9%), loss of employment (36.6%), harassment and threats (24.6%), violence (11.2%), and various other forms of ostracization. Around half of the adults experienced internal stigma and 5.1% associative stigma. About 4.0% reported that they had at least one child who had been denied, suspended or prevented from attending an educational institution in the previous 12 months. About 10.3% of the respondents did not wish to disclose their HIV status to their children for fear of further stigma, discrimination and potential harm to the family’s reputation.

However, there is currently no research about stigma experienced by children in Cambodia. This study evaluated the perceived stigma experienced by children living with HIV attending the National Pediatric Hospital of Cambodia.

## Methods

### Study site

The study was conducted from February 15 to July 30, 2007 in the HIV clinics of the National Pediatric Hospital (NPH) of Phnom Penh during the children’s routine visits. In the NPH, ART for children was first started in 2004. Outcomes were excellent although unacceptably high pre-ART mortality and losses to follow-up were described in a retrospective cohort survey of 1168 HIV-positive children less than 15 years of age [20,21].

### Study procedures and questionnaires

Consecutive Cambodian children above 12 months and under 15 years old were included if they were on ART and if consent from the parent or guardian had been obtained. One interview was conducted with the parent/guardian and child together in Khmer language at the NPH. For children younger than 7 years, the parent/guardians were asked questions about the child’s experiences with perceived stigma and ART care and treatment. Children who were 7 years or older were asked to respond to these questions themselves in the presence of their parents/guardians.

A 47-item questionnaire, pre-tested with five Khmer families and revised based on their comments, was used. It included sociodemographic questions about the child and its parents (but not the parents’ age), questions about the vital status of the parents (if available), access to care, compliance with ART by the child, difficulties and any side effects due the medication, problems related to treatment, and attendance at school.

Two questions were asked on knowledge and fear related to the disease. The questionnaire on perceived stigma was adapted from the Jacoby scale, that we used in another survey [22,23]. Questions were specifically chosen to reflect the pediatric context. The 3-item Jacoby scale was adapted into 4 dichotomous items (agree/disagree) in order to easily measure perceived stigma. Before asking the questions related to perceived stigma, the interviewer explained that all questions were related to the child’s current disease. The following questions were asked to the child or the caregiver for children below 7 years. Did the child play with other children? Was the child integrated into the community? Was the child invited by other children? Was the child rejected by others because of the disease? Perceived stigmatization was recorded as positive if any of the questions were answered in the affirmative.

Incomplete adherence to treatment was evaluated by the recall of missed treatment in the preceding 4 days and the preceding 30 days, with both measures dichotomized as 100% vs. <100% adherent.

Data relating to the child’s HIV status, disease history and treatment was retrieved from hospital records after the interview. The child’s health status was confirmed by the physician on duty. The evolution of the child’s health since the onset of treatment was reported by the parents/guardians (improved, deteriorated, stable).

### Definitions

Poverty was defined as earning less than one U.S. dollar per person per day. Single orphans were children whose mother or father had died and double orphans were those who had lost both parents to HIV/AIDS.

### Sample size

Using Stata Version 8 (Stata Cooperation, College Station, TX), a required sample size of 189 people was calculated. As no published data to calculate the sample size was available, we assumed that 50% of children knew about their disease and that half of the sample (40%) would report perceived stigma. To detect a perceived stigma rate between 30 and 40% we calculated that a total of 172 children was needed with 10% precision, alpha = 0.05, power 80%. We added 17 children (10%) for non-exploitable files raising the total number to 189 children.

### Data management and analysis

Data were entered in Epidata freeware (www.epidata.dk, Odense, Denmark) and cross-checked against original data sheets. We used open-ended questions to determine occupations, reasons for missed appointments, difficulties in treatment, etc. All responses were recorded and eventually categorized by common themes. A new variable was created for each common theme using a 0–1 classification and tested in univariate analysis.

Analyses were carried out with Stata software, Version 8 (Stata Corporation, College Station, TX, USA). Chi^2^ or Fisher’s exact test were used to assess associations between categorical variables as appropriate, and Student’s t-test for two normally distributed continuous variables. P ≤ 0.05 was considered significant. Associations between perceived stigma and children’s characteristics were initially measured using bivariate analyses (age over 7 and 10 years, sex, socio-economic conditions, death of one or both parents, schooling, time since diagnosis and ART, tolerance to ART, and adherence to treatment). Multivariate analyses between perceived stigma and children’s characteristics were conducted initially by introducing into the model the variables significantly associated with perceived stigma (those with *p*-values <0.2, Tables 1 and 2). Then, a back-step selection procedure using odd ratios (OR) was used to leave only those with a *p*-value <0.05 in the final model. Secondary multivariate analyses were conducted among children older than 10 years who had more knowledge about HIV-related diseases and psychosocial needs and among children living with parents, or without one or both of them.

**Table 1:** **Socio-demographic characteristics of children on ART and reports of or non report of perceived stigma at National Pediatric Hospital**

	**Perceived stigma**	**No perceived stigma**	**Odd ratio (95% CI)**	**p**	**Total**
	**n = 79 (43.2%)**	**n = 104 (56.8%)**			**n = 183 (100%)**
Females	33 (41.7)	51 (49.0)	0.7 (0.3-1.4)	0.3	84 (45.9)
Median age in years (IQ range)	7.9	6.1	-	0.002	7.0
	[6.0-9.8]	[3.5-9.0]			[2.0-9.6]
**Age over 7 years**	59 (74.6)	54 (51.9)	2.73 (1.4-5.4)	0.001	113 (61.7)
**Live in main city**	53 (67.0)	79 (75.9)	0.6 (0.3-1.3)	0.18	132 (72.1)
Single orphan, mother died	28 (35.4)	36 (34.6)	1.0 (0.5-2.0)	0.9	64 (34.9)
**Single orphan, father died**	39 (49.4)	43 (41.3)	1.3 (0.7-2.5)	0.2	82 (44.8)
Double orphans	21 (26.6)	24 (23.1)	1.2 (0.5-2.5)	0.5	45 (24.6)
Parents with HIV	67 (84.8)	83 (79.8)	1.4 (0.6-3.3)	0.3	150 (81.9)
-Mother with HIV	61 (77.2)	76 (73.1)	1.2 (0.6-2.6)	0.5	137 (74.9)
Parents’occupation					
-Low skill worker (Father)*	49 (62.0)	64 (61.5)	1.0 (0.5-1.9)	0.9	113 (61.7)
-Low skill worker (Mother)	70 (88.6)	93 (89.4)	0.9 (0.3-2.6)	0.8	163 (89.1)
Parents’education (Father)					
Illiterate	11 (13.9)	16 (15.4)	0.8 (0.3-2.1)	0.7	27 (14.7)
**Primary**	22 (27.8)	37 (35.6)	0.6 (0.3-1.3)	0.2	59 (32.2)
Parents’education (Mother)					
Illiterate	23 (29.1)	37 (35.6)	0.7 (0.3-1.4)	0.3	60 (32.8)
-Primary (Mother)	57 (72.1)	79 (75.9)	0.8 (0.3-1.6)	0.5	136 (74.3)
**Living on less than $1** US/day	61 (77.2)	69 (66.3)	1.7 (0.8-3.5)	0.1	130 (71.0)
Social support**	16 (20.2%)	36 (34.6%)	0.4 (0.2-0.9)	0.03	52 (28.4%)
Go to school (over 5 yrs)***	60 (89.5)	51 (73.9)	3.0 (1.0-9.2)	0.01	111 (81.6)
Go to school****	67 (84.8)	69 (66.3)	2.8 (1.2-6.4)	0.004	136 (74.3)
	[30.1-42.8]	[27.8-37.1]			
Time since HIV diagnosis (months)	36.5	32.5	-	0.3	34.2
	[30.1-42.8]	[27.8-37.1]			[30.4-38.0]
Duration of ART (months)	36.6	36.9		0.9	36.8
	[31.1-42.0]	[32.0-41.9]			[33.2-40.4]
Mean CD4 at 12 months (N = 104)	19.4	19.5		0.9	19.5
	[16.9-22.0]	[17.4-21.6]			[17.8-21.1]
Decreased ART adherence	8 (10.1)	9 (8.6)	1.1 (0.3-3.6)	0.7	17 (9.3)

Variables in bold with p < 0.2 were included in the multivariate analyses.

CI: Confidence interval. Numbers and (percentages). Median and (interquartile range), Mean and [95% confidence interval].

*Low skill worklers were: farmers, workers or factory worker, moto-taxi driver, housework.

^**^Mostly transportation fees and food supplements.

***Of 136 children (67 with stigma and 69 no stigma) aged over 5 years eligible for education.

****All children included in the survey.

**Table 2:** **Perception of disease and perceived stigma of children on ART at National Pediatric Hospital**

	**Perceived stigma**	**No perceived stigma**	**p**	**Total**
	**79**	**104**		**183**
	**(43.2%)**	**(56.8%)**		
Knows his disease*	**31 (39.2)**	**30 (28.8)**	**0.1**	**61 (53.9)**
**Afraid of his/her disease**	**23 (29.1)**	**20 (19.2)**	**0.2**	**43 (23.5)**
Stigma				
Because of his/her disease:				
Does not play with others	26 (32.9)	0		26 (14.2)
Not integrated in the area	36 (45.6)	0		36 (19.7)
Not invited by others	34 (43.0)	0		34 (18.6)
Rejected by others	49 (62.0)	0		49 (26.8)

Variables in bold were included in the multivariate analyses. Numbers and (percentages).

*Of 113 children older than 6 years.

### Ethics statement

The study was authorized by the National Pediatric hospital authorities. Ethical approval was granted by the Lao Medical Ethics Committee. The study complied with the Cambodian law on personal protection of PLWHA. Children and parents/guardians were informed about the study in Khmer language and given an information sheet describing the study. Children were included if they consented to participate and if their parents/guardians had given written informed consent. Confidentiality was guaranteed and interviews were conducted in a private room. Data was recorded anonymously.

## Results

There were no refusals to participate to the survey. A total of 183 children were enrolled in the survey and were interviewed with the help of 138 parents (75.4%) and 45 guardians (24.6%). Their social and treatment characteristics and their relationships with perceived stigma are shown in Table 1. Of 183 children, 101 (55.2%) had lost one parent and 45 had lost both (24.6%).

A total of 44 (24.0%) children was cared for by their grandmothers, 30 (16.4%) by some other member of the family, and 10 (5.5%) children by a non-governmental organization (NGO).

A total of 52 (28.4%) families and their children received some social support; 37 (20.2%) children received help for transportation to hospital, another 12 (6.6%) for food and transportation and 3 (1.6%) of them global assistance from an NGO.

All but two children were on first-line ART (152, 84.0% on 3TC + D4T + NVP; 23, 12.6% on 3TC + ZDV + NVP; 8, 4.3% on 3TC + D4T + EFV). Another two were on second-line ART (kaletra + DDI + ABC). The mean duration on ART was 36.8 [33.2-40.4] months. Median CD4 percentage was 9.0% (95% CI: 8.26-10.3) at initiation of ART, and 16.4% (95% CI: 15.0-17.7) at 6 months (n = 156), 19.5% (95% CI: 17.9- 21.1) at 12 months (n = 104). The time since diagnosis, duration of ART of median CD4 at initition of ART and after 12 monts were not significantly associated with perceived stigma.

According to medical records and the ward physician, three children (1.6%) were in a severe condition (life threatening) and 10 (5.5%) children had opportunistic infections.

Only seven (3.8%) children took their dosage by themselves. Of 183 children, 18 (9.8%) had minor side effects (4 nausea, 5 rash and the others either fever or unspecific pain); 40 (21.9%) spat up their drugs: 8 frequently (4.4%); 32 rarely (17.5%). A 100% adherence to ART was reported among 166 children (90.7%). Decreased adherence was reported by 11 (6.0%) for the 4-day and 8 (4.4%) for the 30-day measures. Only 3 (1.6%) had missed appointments at the hospital (2 forgot, one did not want to go). Decreased adherence was not associated with perceived stigma (The estimated power on this variable was only 32%). The main difficulties regarding treatment were related to: 1) going to the hospital (113, 61.7%), 2) swallowing drugs (23, 12.6%), 3) taste of the drugs (14, 7.6%), 4) lack of money and transportation means (13, 7.1%), and 5) child depression (3, 1.6%).

A total of 166 (90.7%) children went to kindergarten or school. Among 136 children over 7 years, 7(3.8%) reported not going to school due to perceived stigma.

Of 113 children older than 6 years, 61 (53.9%) reported knowledge of their HIV status (Table 2). A total of 43 (23.5 %) children were fearful of their disease. Fear of the disease was strongly associated with children who answered that they knew the name of their disease (1, 0.8% versus 42, 97.7%; p < 0.000). The precise reason for fear was not assessed by the interviewer.

A total of 53 children (28.9%) were over 10 years old and 28 (52.8%) reported perceived stigma.

Of 183 children, 79 (43.2%) reported perceived stigma (Table 2). Because of the child’s current disease, 49 (26.8%) were rejected by others, 36 (19.7%) reported lack of integration into their community, 34 (18.6%) were not invited by others to join activities, 26 (14.2%) did not play with others. Among those aged over 7 years who attended school and answered the questions (n = 103), 43 (41.7%) complained of being rejected because of their disease. The HIV status of parents, the death of one or both parents, the knowledge or fear of the disease were not significantly associated with perceived stigma.

The multivariate regression analyses showed that receiving some kind of social support was an independent factor associated with a decreased risk of perceived stigma (OR: 0.44, 95% CI 0.2-0.9), while attending school (OR: 3.9, 95% CI 2.0-7.9), or living on less than one dollar a day (OR: 2.2, 95% CI 1.1-4.5) was associated with an increased risk of perceived stigma (Table 3).

**Table 3:** **Risk factors associated with perceived stigma in children, multivariate analysis, National Pediatric Hospital, Cambodia**

**Stigma**	**Odds ratio**	**95% CI.**	**z**	**P > z**
Social support	0.44	0.2-0.9	−2.23	0.026
Go to school	3.99	2.0-7.9	3.95	0.000
Less than 1 US$/day	2.23	1.1-4.5	2.22	0.027

CI: Confidence interval.

Variables <0.02 in bold in Tables 1 and 2 were introduced into the model. ORs are only shown for variables forced into the model and those remaining in the model.

For children over 10 years, the final multivariate regression analyses showed that only poverty (OR: 4.7, 95% CI 1.2-17.6, p = 0.02) was independently positively associated with perceived stigma. However, receiving support (OR: 0.2, 95% CI 0.8-1.2, p = 0.09) showed a trend of negative association with perceived stigma.

Receiving support was negatively associated with perceived stigma if the father (OR: 0.2, 95% CI 0.09-0.6, p = 0.003) or mother was dead (OR: 0.3, 95%CI 0.1-1.0, p = 0.07).

For double orphans, the final multivariate analysis showed that having one parent with known HIV (OR: 4.2, 95% CI 1.0-16.3, p = 0.03) was independently positively associated with perceived stigma, and living in Phnom Penh (OR: 0.2, 95% CI 0.6-0.9, p = 0.03) was negatively associated with perceived stigma.

## Discussion

Surveys assessing stigma or stigma-related interventions in children living with HIV are uncommon in Asia [4,13,24-27]. This is the first study on this subject in Cambodia. This study was restricted to perceived stigma, and shows that 43.2% of children experienced HIV-related perceived stigma. This survey shows that perceived stigma and fear of being stigmatized are important social problems for children on ART in Cambodia.

However, despite the alarming rate of stigmatisation it is encouraging that most children (81.6%) of a school going age, could attend an educational institution. Only a minority 7 (5.14% ) of interviewees stated that perceived stigma was the reason for being excluded from school. This figure is consistent with the recent *People Living with HIV Stigma Index Cambodia* study which reported that 4% of their children were excluded from school because of stigma and similar to figures described in 25 provinces of China [28]. This is consistent with UNAIDS report than showed that as a consequence of stigma, some HIV-infected children still face restricted access to school while the majority face some type of exclusion [1].

Of concern is the fact that going to school was one of three main factors associated with perceived stigma. In fact, young children living with HIV are known to be prone to rejection and are perceived as different but few studies have explored stigma among schoolchildren [29]. In addition, fear of transmission, and misconceptions of the disease have been reported as reasons for stigmatization at school [30]. The results of our study suggest to further address enacted stigma and coping mechanisms at school; two issues that were not addressed in the study. Increasing children’s knowledge and dispelling myths about HIV/AIDS may reduce stigma and discrimination at school. In Thailand children’s attitudes have become more supportive of their HIV/AIDS affected peers after the implementation of HIV/AIDS prevention education at schools [31].

However, it has been suggested to start intervention early in primary school before the challenging period of adolescence [32]. Interestingly, among the 53 children above 10 years, schools did not appear a significant factor in perceived stigma but this could be linked to our limited subsample.

Our findings show that poverty and support were diametrically associated with higher or lower perceived stigma. Poverty has been frequently associated with stigma [25,27]. In fact, privacy about a child’s HIV status [10] is virtually impossible when living in poor conditions (10 were living in slums). Hence it is impossible to avoid disease disclosure to neighbours and as a consequence stigmatization is an issue highly feared by the families. The issue of poverty is critical to mitigating the negative impact of HIV and AIDS on children and households but “this area clearly needs intervention” [13]. Our results suggest developing an intervention to decrease stigma in children by targeting particularly underprivileged communities where HIV-affected children live. For affected children some interventions such as psychosocial support, education in coping strategies, and peer support groups have been proposed and have demonstrated a significant reduction in HIV-related stigma [33]. For the community, emphasis has been on education campaigns through provision of factual information about children who suffer from HIV/AIDS. Since the survey, an intervention including a psychosocial support, peer groups and HIV education was developed on a small scale for children attending the NPH and showed promising results but had to be discontinued for financial reasons (HB, personal communication).

The average adherence to treatment was over 90%. Unlike adults, stigma did not negatively influence adherence to ART [8]. This can be explained by the fact that treatment was mostly given by adults to 96.2% of the children and a possible result of the communication strategies of the Cambodia’s human immunodeficiency virus program (NCHADS, National Center for HIV/AIDS, Dermatology and STD, Sexual Transmissible Disease) [17].

A group of three children reported depression as the cause for missed hospital appointments. In fact the psychosocial dimensions of children’s depression and psychological troubles were not specifically assessed in this survey. These preliminary results point out the necessity for evaluating psychological needs further and probably to improve the offer of an integrated psychological care at children’s HIV clinics [27]. There is limited experience on this issue in Cambodia which needs to be scaled up. The vulnerability of HIV/AIDS affected children to psychological disorders of various degrees has been previously shown, but rarely in Asia [27,34]. Internal stigma may be associated with increased stress and psychological problems in children [35]. It was also shown that experiencing stigma and the children’s perception of the public’s stigma against PLWHA are generally stronger predictors of psychological problems than their own feelings or attitudes towards PLWHA [36]. Offering integrated psychological care will require the training of psychologists, their integration into health facilities and the development of psychosocial programs.

Unlike other children who had one parent dead, the multivariate analysis showed that living in Phnom Penh (OR: 0.2, 95% CI 0.6-0.9, p = 0.03) was negatively associated with perceived stigma for double orphans. To answer this issue the Cambodia’s human immunodeficiency virus program launched the 3.0 framework which target zero discrimination. This included systematic linkages between the community and health facilities through the involvement of existing home based care teams and health centers. This is considered to contribute greatly to the reduction of stigma and discrimination by increasing awareness that AIDS is no longer a death sentence [37].

Despite 45 (24.5%) children being orphans (both parents), only 10 (5.46%) of them were living in an institution. The majority were being cared for by family members. This result raises the question regarding the relationship between care providers, families and perceived stigma. In fact, the limited sample size did not allow comparing perceived stigma differences between institutions or community-based caregivers and families. A survey done in five low income nations, including Cambodia, showed that HIV-related stigma of orphans and abandoned children was lower among institution-based caregivers than among those who were community-based. It was also higher amongst older but lower educated caregivers [38]. The role of families as a barrier to stigma was not documented in this multicentric survey. We previously showed that stigma was lower among families with a patient suffering from other stigmatized diseases (such as epilepsy) than among the general population [35]. The contribution of families in decreasing stigma and misconceptions throughout the population is still to be addressed.

### Perspectives

Our findings show that basic social support is a protective factor against children’s perceived stigma. This corroborates a few studies suggesting that social and psychological support can decrease the burden of stigma in children, including depression [14,25]. We provided some discussion regarding target and priorities and future researches in the previous paragraph. The literature shows that interventions to reduce AIDS stigma are likely to be more effective if they are context-specific and sensitive to the prevailing sociocultural and economic environment of each country [9]. Several community-based interventions with multiple activities, including awareness raising, sensitization and community participation and interaction, and peer support groups demonstrated significant changes in developing countries [1,39]. However, they rarely target children in their community and are often implemented on a small scale [1]. They also rarely address the impact of perceived stigma and global stigma on children’s adherence, clinical outcomes, development and quality of life. Our results and the literature suggest that in Cambodia, increasing social and psychosocial interventions, addressing stigma in schools and among populations living below the poverty line, should be considered [1,3,40]. A recent review suggests that combining various strategies to reduce stigma may have greater impact in enhancing participants’ understanding about the effect of HIV/AIDS stigma in at-risk populations [13,41].

### Limitations of the survey

This survey has several limitations. The limited sample size, the restriction of the study to perceived stigma and use of a non-validated scale for perceived stigma were limits of the survey. The questionnaire and scale were pre-tested only for accuracy and comprehension. We adapted the Jacoby scale that was validated for another disease. Its advantage was to allow a rapid assessment, to be adapted to children and that we had some experience with it. This was considered important due to the time frame and conditions of the study and also in order not to impact on the children own perception of his/her disease.

Hence, the questionnaire was not able to describe all components of stigma or describe stigma experiences. This brief and preliminary investigation of perceived stigma in children will benefit from further in-depth documentation [42-44].

Additionally, perceived stigma was possibly underestimated by asking the questions of caregivers for orphaned children below 7 years. Caregivers’answers about the children’s feelings might be regarded as approximate and not completely reflecting the opinion of the children.

Due to time and budget constraints it was not possible to design a study with a representative population. This is a limitation that was possibly minimized by the fact than nearly half of the children (39.8%) came from various provinces.

The time frame of the study, which was conducted some time ago, could also be considered a limitation. In fact, according to Cambodian pediatricians and patients’ families, fear of the stigmatization remains a major problem for children in 2014 (Personal communications to H. Barennes and Ung Vibol). This issue was recently documented, for adults, in the *People Living with HIV Stigma Index Cambodia* study [19]. A majority of parents do not wish to disclose their HIV status for fear of further stigmatization, discrimination and potential harm to their families.

## Conclusion

Perceived stigma in pediatric ART patients remains a significant issue in Cambodia. Social support for children helps them to cope with perceived stigma. Community-wide stigma reduction program and psychological support should be part of the holistic care efforts for children living with HIV. Education programs should focus on prevention of stigma at school, in hospitals and in underprivileged communities in Cambodia. Further research is required to better understand other aspects of stigma within the children’s community and to identify key opinion groups and effective strategies that would help decrease stigma among children living with HIV.

## Abbreviations

ART: Antiretroviral therapy
CI: Confidence interval
HIV: Human immunodeficiency virus
OR: Odds ratio
NPH: National Pediatric Hospital
NCHADS: National center for HIV/AIDS, Dermatology and STD, Sexual transmissible disease
PLWHA: People living with HIV/AIDS
